# Continuities and changes in spatial patterns of under-five mortality at the district level in India (1991–2011)

**DOI:** 10.1186/s12942-018-0159-3

**Published:** 2018-11-15

**Authors:** Akansha Singh, Bruno Masquelier

**Affiliations:** 1 Centre de recherche en démographie, Université Catholique de Louvain, Louvain-la-Neuve, Belgium; 2 Institut National d’Etudes Démographiques (INED), Paris, France

**Keywords:** Under-five mortality, India, Geographically weighted regression, District

## Abstract

**Background:**

India has the largest number of under-five deaths globally, and large variations in under-five mortality persist between states and districts. Relationships between under-five mortality and numerous socioeconomic, development and environmental health factors have been explored at the national and state levels, but the possible spatial heterogeneity in these relationships has seldom been investigated at the district level. This study seeks to unravel local variation in key determinants of under-five mortality based on the 1991 and 2011 censuses.

**Methods:**

Using geocoded district-level data from the last two census rounds (1991 and 2011) and ordinary least squares and geographically weighted regressions, we identify district-specific relationships between under-five mortality rate and a series of determinants for two periods separated by 20 years (1986–1987 and 2006–2007). To identify spatial groupings of coefficients, we perform a cluster analysis based on *t*-values of the geographically weighted regression.

**Results:**

The geographically weighted regression analysis shows that relationships between the under-five mortality rate and factors for socioeconomic, development, and environmental health factors vary spatially in terms of direction, strength, and extent when considering: female literacy and labor force participation; share of scheduled castes and scheduled tribes; access to electricity; safe water and sanitation; road infrastructure; and medical facilities. This spatial heterogeneity is accompanied by significant changes over time in the roles that these factors play in under-five mortality. Important local determinants of under-five mortality in 2011 were female literacy, female labor force participation, access to sanitation facilities and electricity; while the key local determinants in 1991 were road infrastructure, safe water, and medical facilities. We identify six different clusters based on geographically weighted regression coefficients that broadly encompass the same districts in both periods; but these clusters do not follow the regional boundaries suggested by the previous studies. In particular, the high mortality states of India that are often typically classified as high focus states were classified into three different clusters based on the relationship of the factors associated with under-five mortality.

**Conclusion:**

This study demonstrates the utility of combining geographically weighted regression and cluster analyses as a methodological approach to study local-level variation in public health indicators, and it could be applied in any country using aggregate-level information from census or survey data. Identifying local predictors of under-five mortality is important for designing interventions in specific districts. Additional reduction in under-five mortality will only be possible with intervention programs designed at the local level, which take into consideration local level determinants of under-five mortality.

## Introduction

Reducing the under-five mortality rate (U5MR) by two-thirds (between 1990 and 2015) was a key Millennium Development Goal, and tracking progress in this goal has spurred a flurry of methodological developments in generating robust estimates of U5MR at the national scale [1–3]. The focus on levels and trends in U5MR through national averages may have somewhat obfuscated differential progress within countries. The Sustainable Development Goals (SDG) agenda marks a shift in the fight against child mortality, since increased attention is now devoted to closing equity gaps while reducing the overall level of mortality. The SDG’s U5MR target for 2030 is to end preventable deaths of newborns and children under five years of age. All countries should aim to reduce neonatal mortality to at least as low as 12 deaths per 1000 live births, and U5MR should be at least as low as 25 deaths per 1000 live births [4]. With this new SDG agenda, it is crucial to develop subnational estimates to ensure that no one is left behind, especially in large and decentralized countries such as India.

With a population of 1.3 billion and U5MR still estimated at 43 deaths per 1000 live births in 2016, India has the highest number of child deaths in the world. In 2016, an estimated one million children died in India [5], corresponding to about one-fifth of the global number of under-five deaths. The country failed to achieve the MDG 4 target, despite reducing U5MR by 64% between 1990 and 2015. There are also large variations in U5MR between states [6]. According to the Sample Registration System (SRS) data for the year 2015, the U5MR of Assam in Northeast India was 62 deaths per 1000 live births, which is more than four times higher than that of Kerala in South India, where the U5MR was estimated at 13 per 1000 [7]. Numerous studies have highlighted higher mortality rates in the northern states [8–13] compared with states in the southern and western parts of the country. A north–south divide contrasts with inequalities in adult mortality, where the largest differences are observed between eastern India and western India [9]. Some studies have also demonstrated that child deaths are highly clustered and that improvements in U5MR have been uneven, with certain hotspots still bearing the brunt of under-five deaths, especially Assam, Madhya Pradesh, Rajasthan, Uttar Pradesh, and Orissa [10, 11]. In this context, achieving the SDG target will require identifying areas that are falling behind and targeting them with specific interventions that will ideally reach beyond the state level. The lowest level of disaggregation in India is the district, which constitutes the basic administrative unit. Yet, relatively few studies have examined the levels and determinants of U5MR at this level in India. Numerous studies have analyzed cause-specific mortality changes [12], explored U5MR risk factors at the national or state level [13], or they have classified India into broad geographic regions [8]. Studies that disaggregate their estimates at the district level have focused on developing accurate mortality estimates [14] or investigating sex differentials [15]. However, efforts to identify determinants of U5MR at the district level are often restricted to selected states only [10] or to a single census. The work of Bhattacharya [16]—which refers to the period before 2000—and that of Bhattacharya and Chikwama [17]—which refers to the period until 2001—are two noticeable exceptions. These last two studies suggest that variables related to modernization and economic development (such as access to medical facilities and access to urban areas through paved roads) together accounted for more than 40% of the decline in U5MR during the 1981–1991 period. By contrast, variables related to the agency of women—such as literacy and their participation in the labor market—accounted for only 6% of this decline. These previous studies also indicated that while mortality declined overall, inequalities in child survival increased between 1981 and 2001 [16]. While better access to medical facilities and safe drinking water contributed to reducing inequalities, other dimensions of structural change—such as infrastructure development, female literacy, and socio-cultural variables—showed differential progress and consequently contributed towards increased inequality in U5MR across districts [17]. However, these analyses capture only the overall effects of specific determinants on U5MR. In this paper, we build on these works but additionally investigate the spatial heterogeneity in the relationships between district-level U5MR and a set of significant socioeconomic, development, and environmental health factors in India, all of which we have based on census data from 1991 and 2011. We focus less on differences *between* regions and more on differences *within* regions.

We aim to test whether the associations between U5MR and major determinants vary locally. If this is the case, a single global model may not readily summarize these relationships. For example, female literacy could shape inequalities in U5MR in some specific areas where there are still important gaps in education, but it could play only a minor role when a vast majority of women are literate. We assess spatial heterogeneity in U5MR and the relationships of its associated factors using geographically weighted regression (GWR).

This study is novel because it not only tests the spatial heterogeneity in the relationship between U5MR and its associated factors, but it also examines the temporal variations in such relationships for the same area. By comparing the 1991 and 2011 censuses, we are able to identify the underlying factors that drive mortality patterns at the district level and to capture changes over time in the magnitude and direction of relationships between key determinants and U5MR at the local level. Previous studies using global regression models [16, 17] indicate that the significant factors related to U5MR variation are not the same over these years. We further test U5MR and its predictor’s relationship for regionality by applying cluster analysis to the GWR results. Clustering districts based on the local regression coefficients from GWR analysis identifies regions with similar relationships. Several previous analyses have found geographic variability in regression relationships [18–20], but these analyses are based on predefined regions, and it is possible that these definitions influence the chances of finding regional variability. Regional boundaries defined using cluster analysis are data-driven boundaries rather than anything that is predefined geographically or based on any single indicator.

## Methods

### Data

Censuses are conducted in India approximately every ten years, with the last three censuses having been conducted in 1991, 2001 and 2011. For this analysis, we used district-level data from two rounds of census data (1991 and 2011) provided by the Registrar General of India to estimate U5MR and build a set of explanatory variables.

### Outcome variable

Our outcome measure of U5MR is the probability of a newborn dying before reaching age 5 (_5_q_0_) if he or she was exposed to the mortality rates of the period under consideration. All women of childbearing age reported on their total number of children ever born and children surviving in the 1991 and 2011 censuses. This information was the basis for applying a standard indirect demographic method to estimate mortality, referred to as the Brass method [21]. Using model-based coefficients to account for variations in the age pattern of fertility and mortality, this method converts the proportion of dead children born to women in each five-year age group into estimates of the probability of dying by exact ages of childhood, and it calculates the number of years before the survey date to which the estimates refer. The indirect method, described in detail elsewhere [22], provides one estimate of U5MR for each age group of women. Reports from the youngest women aged 15–19 typically provide mortality rates referring to approximately one or 2 years before data collection and reports from the oldest women refer to a more distant past, sometimes more than 15 years before data collection. For this analysis, we retained the average of estimates derived from two age groups of women (25–29 and 30–34 years old) to get one final estimate per district for each census. We discarded reports from younger women aged 15–24 because first-born children and children born to young mothers are disproportionately represented in these reports, thus introducing upward biases in mortality rates [22]. We also discarded reports from women older than 35 in order to limit biases due to more frequent omissions of deceased children among older women and due to errors in recalling events that occurred in the more distant past. The reference dates for _5_q_0_ estimates for the 2011 and 1991 censuses are, respectively, 2006–2007 and 1986–1987.

### Explanatory variables

The set of explanatory variables considered in this study is also measured at the district level and constructed from tabulated census data (Table 1). Women’s agency, a key determinant of U5MR, is captured through female literacy among adults aged 15–49 years and through female labor force participation in the age group of 15–59 years. Social variations are reflected in the percentage of females belonging to scheduled castes and scheduled tribes. Scheduled tribes and castes are officially designated groups of disadvantaged people, comprising 8.6% (when considering tribes) and 16.6% (when considering castes) in the 2011 census. The level of the district’s economic development is captured through the percentage of villages with access to *pucca roads* (paved roads) and the urbanization level of the district. The relatively healthy character of the environment is reflected in the percentage of villages with access to medical facilities and the percentage of households with access to sanitation facilities and safe water.

**Table 1:** Description of variables used in this study

Under-five mortality rate (_5_q_0_)	Probability of dying between birth and the exact age of 5
Female literacy	Percentage of women aged 15–49 in the district who are literate
Female labor force participation rate	Percentage of women aged 15–59 in the district who are currently at work
Female scheduled caste and tribe	Percentage of women belonging to a scheduled caste or scheduled tribe in the district
Access to sanitation facilities	Percentage of households in the district with access to toilet facilities
Access to safe water	Percentage of households in the district with access to safe water from tap, hand pump and tube well (treated and untreated)
Road infrastructure	Percentage of villages in the district approached by pucca (paved) roads
Access to medical facilities	Percentage of villages in the district having medical facilities
Urbanization	Percentage of the population in each district living in urban areas
Access to electricity	Percentage of households in each district with access to electricity

### Analysis

Some district boundaries have changed between 1991 and 2011. For this study, however, the districts in 1991 were considered as the reference, and districts that had been separated in recent years were merged into the original district. In total, we retained 449 districts. We excluded districts from Jammu and Kashmir, Lakshadweep, and Andaman and Nicobar Islands for the following reasons. One, the census was not conducted in Jammu and Kashmir in 1991. Two, Lakshadweep, Andaman, and Nicobar are islands and it is therefore difficult to define their spatial neighbors and calculate the bandwidth in GWR.

To capture the pattern and magnitude of spatial clusters, we first explored the significant local spatial clusters/outliers of U5MR rates using local indicators of spatial association (LISA) statistics [23]. Areas identified as “high–high” refer to districts with above-average U5MR and which also share boundaries with neighboring districts that have above-average U5MR. “High-low” areas point to districts with above-average U5MR and which are surrounded by districts with below-average values. The “high–high” areas are also often referred to as “hot spots”, whereas the “low–low” are referred to as “cold spots”. Spatial dependence in the district-level estimates of each indicator was assessed using Moran’s I Index (Global) value. In general, a Moran’s Index value near +1 indicates clustering, while an index value near − 1 indicates dispersion.

Ordinary least squares (OLS) models were used to obtain coefficient estimates at the global level. The OLS models also helped identify the covariates by means of a stepwise approach before moving on to the GWR. The stepwise regression approach selected variables in the OLS model for the 2011 census and these same variables were retained for the 1991 census. Further multicollinearity tests were performed on the final set of variables. The multicollinearity was assessed through variance inflation factor (VIF) values, and VIFs greater than 10 indicated multicollinearity [24]. This final list of selected variables was retained for both the OLS and GWR analyses. To explore the varying relationships between U5MR and socioeconomic predictors, we used GWR models. The bandwidth size was determined by minimizing the Akaike Information Criterion (AIC). Model comparisons between the OLS and GWR models were conducted using the AIC with a correction for finite sample sizes (AICc). One of the advantages of GWR modeling is that researchers can map the local coefficients as well as *R*^2^ in order to better identify spatial heterogeneities [25].

Following previous studies [26], the basic equation of the GWR model is expressed as:

1 $$y_{i} = \beta_{0i} \left({u_{i},v_{i}} \right) + \sum_{n = 1}^{k} \beta_{ni} \left({u_{i},v_{i}} \right)x_{ni} + \varepsilon_{i}$$

where *y*_i_ refers to the U5MR in the district *i*; (*u*_*i*_, *v*_*i*_) denotes the coordinates of the centroid of district *i*; *β*_0i_ is the local intercept for the district *i*; and *β*_ni_ is the local coefficient for predictor *n* for district *i*. This GWR model was run separately for each census (1991, 2011). In GWR models, the regression coefficients are estimated for each district independently by applying location-specific weighting schemes; therefore, there are as many “local” regression models as there are observations [27]. In matrix form, the vector of local coefficients is estimated as:

2 $$\beta_{i} = \left({X^{\prime} W_{i} X} \right)^{- 1} X^{\prime} W_{i} Y$$

where *X* is the matrix of independent variables, and *Y* is the vector of dependent variables. The estimator in Eq. (2) is a weighted least squares estimator where the weights vary according to the location point of *i*. There are a variety of weighting schemes available to choose for researchers [26]. We chose the Gaussian weights and their bi-square variations, which are the most commonly used options. Thus in Eq. (2), *W*_*i*_ is an n × n diagonal matrix with the *j*-th diagonal element equal to $\left[{1 - (d_{ij}/b)^{2}} \right]^{2}$ if *d*_*ij*_ < *b* and zero otherwise. *d*_*ij*_ refers to the Euclidean distance between location *i*, where the parameters are estimated, and a specific point in space *j* at which data is observed [26]. The parameter *b* is the bandwidth size (i.e. the distance between each observation and its neighboring locations specified by the spatial weights). Further, a spatial non-stationarity test was conducted to test the significance of spatial variability in the local parameter estimates. This was performed using a test developed by Leung and colleagues [28].

Finally, to examine whether there were spatial groupings of coefficients from the GWR and to facilitate the interpretation of the GWR results, we applied cluster analysis to the *t*-values (that is, the local regression parameters divided by the standard errors of the estimates), which were obtained from the GWR model. We use local *t*-coefficients instead of the beta coefficients because the former denote both the direction and effect size of the relationships [29, 30]. K-means clustering was used to get the final number of clusters to be retained. The optimal number of clusters for the K-means method is chosen based on the Elbow method described elsewhere [31]. It is worth noting that these clusters form areas that are not defined by state boundaries and reflect specific patterns of variations in U5MR. Nor do they coincide exactly with regions highlighted in previous studies on India. Simple OLS regression was then performed in each cluster to study the relationships that socioeconomic and environmental factors have with U5MR in these specific clusters. All analyses were performed with the R statistical software.

## Results

**Fig. 1:**
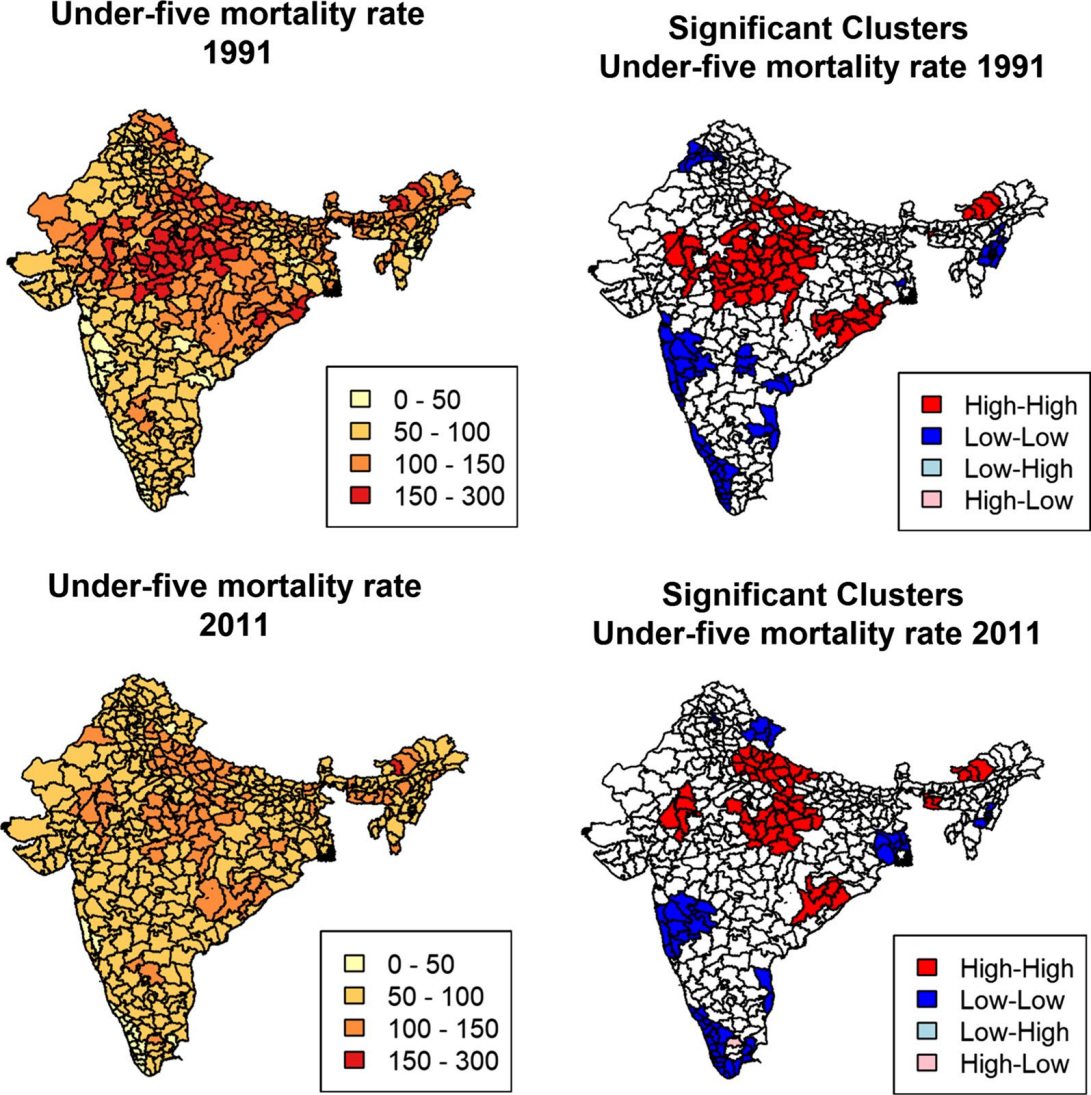
Under-five mortality rates (deaths before age 5 per 1000 live births) and local clusters at the district level in India, 1991 and 2011 censuses

U5MR has declined almost everywhere over the past 20 years, from the period 1986–1987 to 2006–2007, but there are large variations in the pace of decline (Fig. 1). According to the 1991 census, most 1986–1987 U5MR estimates for the districts in the south of the country varied between 50 and 100 under-5 deaths per 1000 live births, and a few districts had less than 50 under-5 deaths per 1000 live births in the south-west of the country (in Maharashtra, near Mumbai). By comparison, according to estimates at the national level that were developed by UN IGME, the U5MR was estimated at 141 per 1000 in 1986 [5]. U5MR was higher than 100 per 1000 live births in most districts in the northern region, especially in states such as Madhya Pradesh and Uttar Pradesh. There were only modest changes in the geography of U5MR from 1986–1987 to 2006–2007, with a clear cluster of high U5MR districts remaining in the center of the country and low U5MR pockets persisting in the south. In estimates derived from the 2011 census, 22 districts had a U5MR below 50 per 1000 while 333 districts managed to contain their U5MR to between 50 and 100 under-5 deaths per 1000 live births. By comparison, the UN IGME estimate for 2006 was 71 at the national level [5].

The LISA cluster maps presented alongside the map of overall U5MR display four types of geographical clustering for U5MR. These maps indicate persistent geographic clustering of high U5MR in Central India and parts of the east. On the other hand, the maps also show a few cold spots with substantially lower U5MR in South India and in a few districts of Northeast India. The number of districts falling into high–high clusters decreased significantly from 1991 to 2011. Quite surprisingly, there were no high-low or low–high clusters in 1991, and only one high-low cluster in 2011. Table 2 shows values of Moran’s I statistic, which is a measure of the spatial autocorrelation among the neighboring values [23]. Moran’s I statistics for all the variables are relatively high, suggesting strong spatial patterns for both dependent and independent variables.

**Table 2:** Moran’s I test statistics for the different variables under study

Censuses	1991	2011
Under-five mortality rate	0.64***	0.60***
Female literacy	0.68***	0.68***
Female labor force participation rate	0.72***	0.68***
Female scheduled caste and tribe	0.55***	0.55***
Access to sanitation facilities	0.38***	0.70***
Access to safe water	0.60***	0.63***
Road infrastructure	0.70***	0.64***
Access to medical Facilities	0.64***	0.60***
Urbanization	0.13***	0.33***
Access to electricity	0.64***	0.77***

*p < 0.05; **p < 0.01; ***p < 0.001

Coefficients from the standard OLS model show the expected relationships between district-level U5MR and socioeconomic, development, and environmental health factors (Table 3). They are also broadly consistent over time. The global model for 1991 explains 46% of the variation in U5MR, and the model for 2011 explains 52% of the variation. According to estimates from the two censuses, female literacy and labor force participation in a district have significant negative relationships with U5MR. As the proportion of women belonging to scheduled tribes and castes increases in a district, U5MR also increases significantly. However, the set of indicators significantly associated with U5MR is not the same across each census. For example, access to safe water at the household level and the percentage of villages with access to paved roads were significantly associated with U5MR in 1991; whereas in the 2011 census, it was households having access to sanitation facilities and electricity and villages having access to medical facilities that were significantly associated with U5MR.

**Table 3:** OLS regression estimates for under-five mortality rate at the district level, in the 1991 and 2011 censuses

	1991					2011				
	Estimate	Std. error	*t*-value	p value	vif	Estimate	Std. error	*t*-value	p value	vif
(Intercept)	172.54	6.56	26.28	0.00***		138.78	7.35	18.88	0.00***	
Female literacy	− 1.08	0.11	− 9.80	0.00***	2.09	− 0.69	0.10	− 6.99	0.00***	2.79
Female labor force participation rate	− 0.34	0.09	− 3.67	0.00***	1.67	− 0.21	0.08	− 2.75	0.01**	1.93
Female scheduled caste and tribe	0.16	0.08	2.11	0.04*	1.54	0.27	0.04	6.06	0.00***	1.69
Access to sanitation facilities	0.07	0.08	0.80	0.43	1.56	− 0.13	0.05	− 2.61	0.01**	2.89
Access to safe water	− 0.18	0.07	− 2.54	0.01*	1.34	0.05	0.04	1.17	0.24	1.34
Road infrastructure	− 0.33	0.07	− 4.57	0.00***	2.09	− 0.04	0.03	− 1.32	0.19	1.56
Access to medical facilities	− 0.03	0.05	− 0.65	0.52	1.57	− 0.09	0.04	− 2.49	0.01*	1.54
Urbanization	− 0.21	0.10	− 2.15	0.03*	1.53	0.18	0.05	3.45	0.00***	1.93
Access to electricity	0.03	0.08	0.44	0.66	2.18	− 0.18	0.04	− 3.89	0.00***	3.10
R^2^	0.46					0.52				
AICc	4296.81					3735.52				

*vif* is variance inflation factor, which quantifies the severity of multicollinearity in an ordinary least squares regression analysis

*p < 0.05; **p < 0.01; ***p < 0.001

**Fig. 2:**
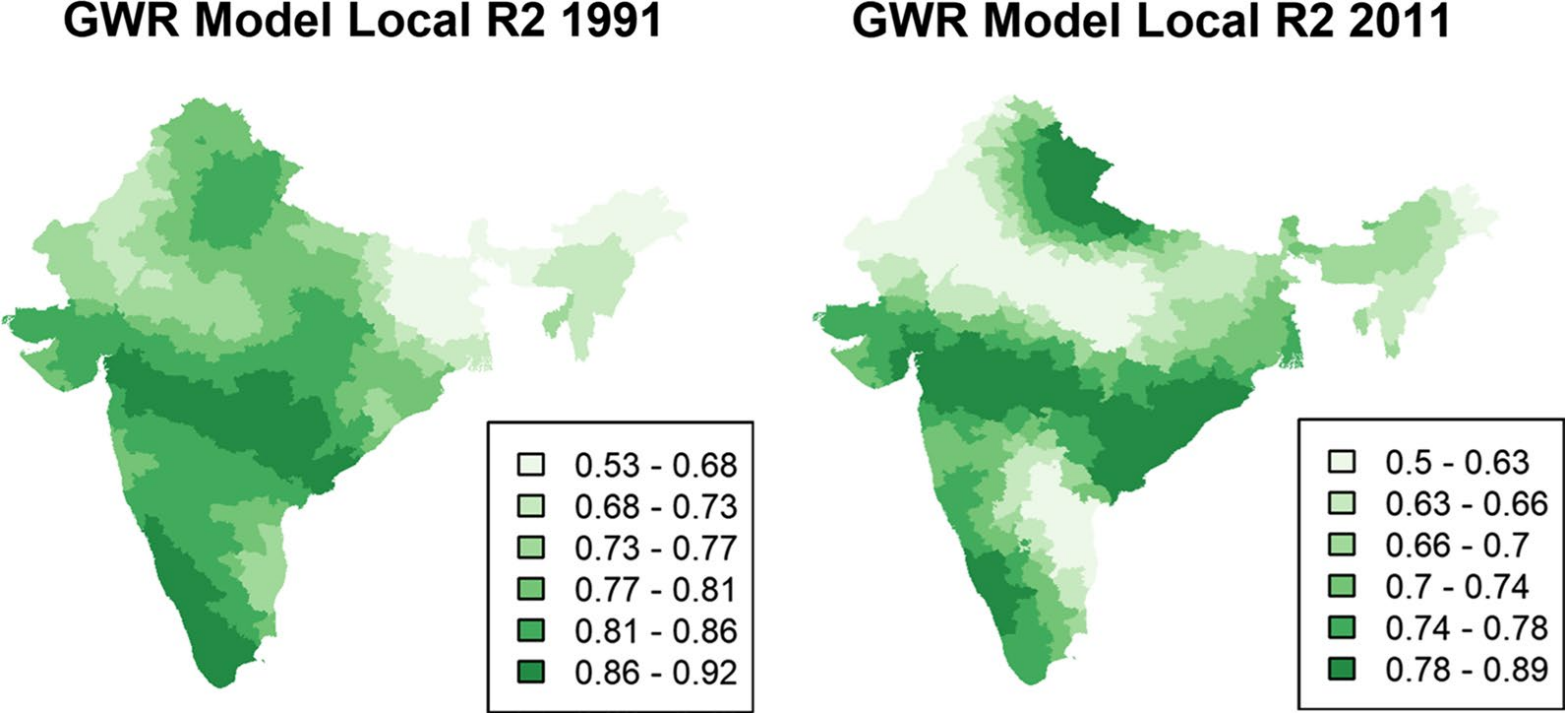
Local R^2^ for GWR

Table 4 provides the estimated coefficients of the GWR model. These coefficients demonstrate how the identified relationships vary from one district to another and to what extent these local relationships remain hidden in the global model presented in the OLS models above. The non-stationarity test results suggest that all the covariates are spatially nonstationary and therefore should be treated as local covariates. This observation holds in both census years. In terms of overall goodness-of-fit when comparing to the OLS model, the Quasi-Global R^2^ is higher than 0.80 for both the GWR models. The AICc of the GWR model is smaller than the OLS model for both census years, suggesting that the former has a better fit than the latter. Local R^2^ maps (Fig. 2) indicate that the GWR model fits well in most places in India, with R^2^ being above 0.50 for all the local models. This value is higher than the R^2^ value of the OLS model. The local R^2^ values for districts from Maharashtra, Karnataka, and Andhra Pradesh are higher than 0.80 for both censuses, suggesting that the current set of indicators capture more than 80% of the variation in U5MR in the districts from these regions (Fig. 2).

**Table 4:** GWR coefficients for under-five mortality rate at the district level, in the 1991 and 2011 censuses

	Min	Q1	Median	Q3	Max
*1991*					
(Intercept)	− 40.09	110.70	152.20	190.30	280.70
Female literacy	− 3.18	− 1.27	− 0.60	− 0.08	0.96
Female labor force participation rate	− 1.19	− 0.45	− 0.12	0.29	1.31
Female scheduled caste and tribe	− 1.04	0.05	0.26	0.49	1.26
Access to sanitation facilities	− 2.18	− 0.32	0.08	0.38	1.07
Access to safe water	− 1.00	− 0.32	− 0.05	0.20	0.81
Road infrastructure	− 1.25	− 0.43	− 0.25	0.13	1.42
Access to medical facilities	− 1.35	− 0.33	− 0.14	0.00	0.43
Urbanization	− 1.16	− 0.37	− 0.09	0.03	0.63
Access to electricity	− 1.13	− 0.50	− 0.26	− 0.03	1.40
AICc	4046.67				
R^2^	0.84				
*2011*					
(Intercept)	34.97	116.70	148.90	161.30	197.40
Female literacy	− 1.24	− 0.77	− 0.56	− 0.34	0.42
Female labor force participation rate	− 1.02	− 0.32	− 0.16	0.19	1.37
Female scheduled caste and tribe	− 0.63	0.00	0.12	0.28	0.94
Access to sanitation facilities	− 0.86	− 0.33	− 0.13	− 0.01	0.27
Access to safe water	− 0.41	− 0.09	0.06	0.23	0.68
Road infrastructure	− 0.60	− 0.09	0.01	0.08	0.30
Access to medical facilities	− 0.43	− 0.16	− 0.06	0.03	0.54
Urbanization	− 0.32	0.01	0.12	0.24	0.73
Access to electricity	− 1.51	− 0.40	− 0.28	− 0.10	0.64
AICc	3617.46				
R^2^	0.80				

Non-stationarity test is significant at p < 0.001 for all variables in the GWR model

**Fig. 3:**
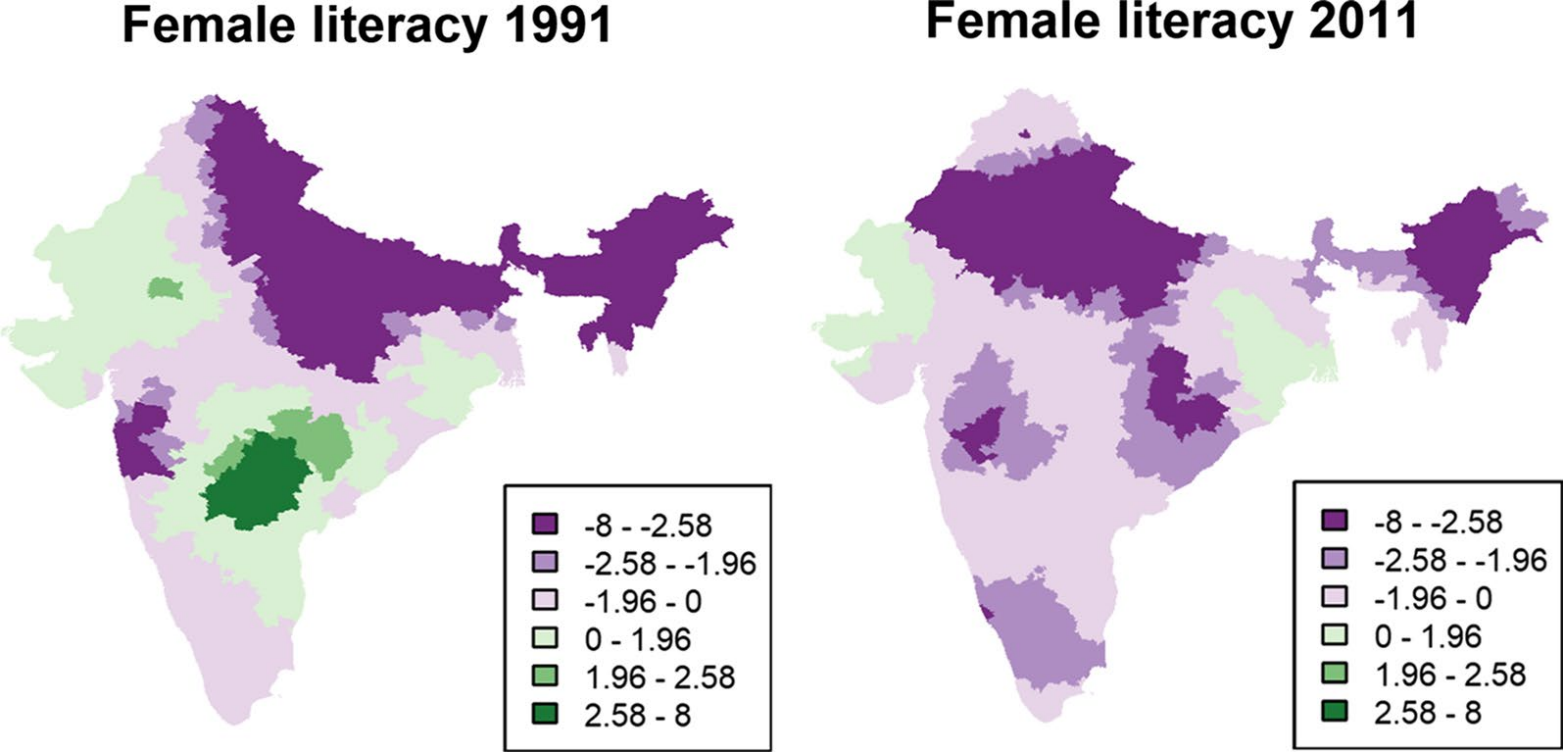
*t*-values of female literacy generated using GWR

More importantly, our results demonstrate that relationships between the local variables and U5MR in India vary substantially. This is illustrated in Fig. 3 for female literacy and in “Appendix 1” for all the other covariates, with the *t*-values of the GWR models included. The green tones represent positive relationships and the purple tones represent negative relationships. Each covariate shows specific spatial variations that cannot be readily summarized in terms of a simple pattern (such as the often-mentioned north–south divide in U5MR). For example, when considering the relationship between U5MR and female literacy, the *t*-values for 1991 are highly significant and negatively associated with U5MR in the northeast, northern, and central parts of India. However, this association was positive and significant for the year 1991 in a series of districts located in the south and in Rajasthan. It is unclear why higher literacy rates could be associated with higher mortality rates in these areas. In 2011, no district showed a significant positive relationship, and the negative association was significant in half of the districts. Similarly, access to sanitation facilities and access to other facilities can be significantly and positively associated in one region and negatively associated in another. Overall, our GWR analysis clearly suggests that the association of each predictor with U5MR varies significantly over space and time. Distinct regions characterized by positive or negative associations can be discerned with coherent evolution over time between the 1991 and 2011 censuses.

**Fig. 4:**
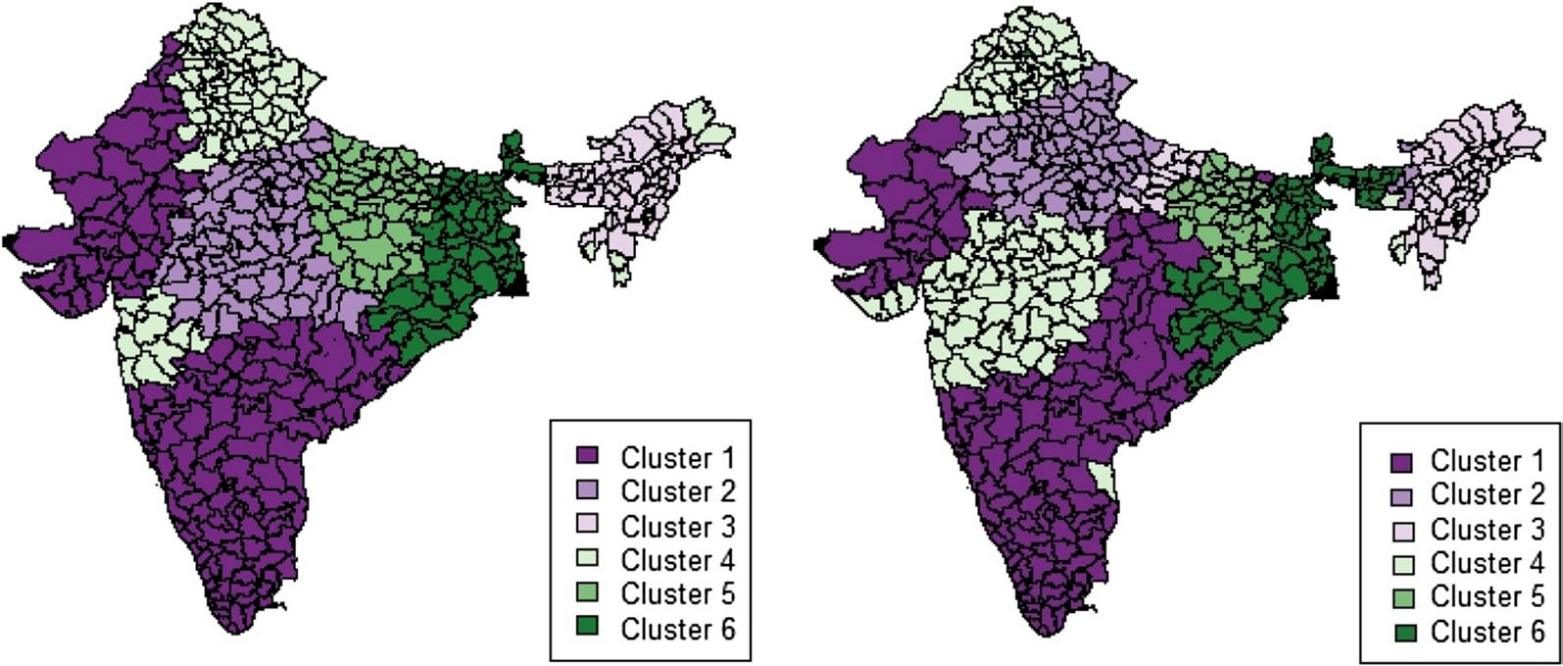
Cluster locations, 1991–2011

For both censuses, an examination of the percentage of variance explained as a function of the number of clusters called for dividing India into six principal clusters (Fig. 4). It should also be kept in mind that the cluster analysis was performed using *t*-value estimates of GWR for each census independently (that is, there is no exchange of information between the 1991 and 2011 censuses). In both censuses, 70% of the 449 districts were part of the same cluster, so these clusters are broadly comparable, even though the shapes and areas covered by each cluster have changed slightly. For example, Cluster 1 is located partly in the south of the country but also with some districts located in the western regions, and it has expanded from 1991 to 2011. For this reason, instead of working with two different sets of clusters, we retained the clusters obtained in 1991 and performed the cluster-specific OLS regressions successively on the 1991 and 2011 data. “Appendix 2” shows descriptive statistics of all the dependent and independent variables considered in this study for each cluster in 1991 and 2011. Comparison of the descriptive statistics for the year 2011 as per the cluster boundaries for 1991 and 2011 suggests that our conclusions do not substantially change even if we retain the same set of clusters for each census year.

To characterize the clusters, we again performed an OLS regression at the district level for each cluster. Table 5 presents the OLS regression results for each cluster in the year 1991 and 2011. “Appendix 2” provides the basic statistics of all independent variables in each cluster. Within the same cluster—the factors significant in both years are not precisely the same. We highlight some peculiarities of each cluster below.

**Table 5:** OLS regression estimates for under-five mortality rate in each cluster, 1991 and 2011

	1991				2011			
	Estimate	Std. Error	*t*-value	p value	Estimate	Std. Error	*t*-value	p value
*Cluster 1*								
(Intercept)	111.22	12.12	9.17	0.00***	110.89	13.33	8.32	0.00***
Female literacy	− 0.43	0.15	− 2.79	0.01**	− 0.51	0.18	− 2.84	0.01**
Female labor force participation rate	− 0.10	0.13	− 0.77	0.45	− 0.08	0.12	− 0.70	0.48
Female scheduled caste and tribe	0.57	0.16	3.46	0.00***	0.36	0.11	3.29	0.00**
Access to sanitation facilities	0.27	0.11	2.33	0.02*	− 0.04	0.09	− 0.46	0.65
Access to safe water	0.17	0.10	1.63	0.11	0.20	0.08	2.43	0.02*
Road infrastructure	− 0.32	0.10	− 3.29	0.00**	− 0.08	0.07	− 1.19	0.24
Access to medical facilities	0.04	0.08	0.50	0.62	− 0.11	0.06	− 1.84	0.07
Urbanization	− 0.29	0.13	− 2.29	0.02*	0.05	0.09	0.56	0.58
Access to electricity	− 0.27	0.13	− 2.16	0.03*	− 0.15	0.13	− 1.12	0.26
Adjusted R^2^		0.56			0.62			
*Cluster 2*								
(Intercept)	250.72	16.46	15.23	0.00***	150.95	22.34	6.76	0.00***
Female literacy	− 0.43	0.38	− 1.14	0.26	− 0.26	0.27	− 0.94	0.35
Female labor force participation rate	− 0.53	0.25	− 2.10	0.04*	− 0.64	0.24	− 2.70	0.01**
Female scheduled caste and tribe	− 0.05	0.26	− 0.19	0.85	0.29	0.17	1.74	0.09
Access to sanitation facilities	− 0.39	0.63	− 0.62	0.54	− 0.51	0.25	− 2.05	0.04*
Access to safe water	− 0.46	0.24	− 1.94	0.06	− 0.25	0.18	− 1.36	0.18
Road infrastructure	− 0.96	0.28	− 3.39	0.00**	0.09	0.11	0.77	0.44
Access to medical facilities	− 0.32	0.12	− 2.61	0.01*	0.05	0.10	0.49	0.62
Urbanization	− 0.20	0.30	− 0.67	0.50	0.16	0.20	0.79	0.44
Access to electricity	− 0.11	0.30	− 0.38	0.71	− 0.13	0.13	− 1.01	0.32
Adjusted R^2^		0.62			0.52			
*Cluster 3*								
(Intercept)	193.58	29.97	6.46	0.00***	176.84	23.57	7.50	0.00***
Female literacy	− 1.95	0.49	− 4.01	0.00***	− 0.71	0.34	− 2.11	0.04*
Female labor force participation rate	− 0.92	0.42	− 2.19	0.03*	− 0.41	0.38	− 1.07	0.29
Female scheduled caste and tribe	0.33	0.26	1.28	0.21	0.38	0.18	2.19	0.03*
Access to sanitation facilities	0.52	0.27	1.92	0.06	− 0.71	0.22	− 3.22	0.00**
Access to safe water	0.43	0.26	1.68	0.10	− 0.04	0.13	− 0.34	0.74
Road infrastructure	− 0.63	0.47	− 1.32	0.19	0.26	0.16	1.64	0.11
Access to medical facilities	0.32	0.43	0.74	0.47	0.01	0.18	0.08	0.93
Urbanization	0.53	0.58	0.93	0.36	0.24	0.29	0.85	0.40
Access to electricity	− 0.91	0.49	− 1.84	0.07	− 0.23	0.22	− 1.06	0.30
Adjusted R^2^		0.49			0.43			
*Cluster 4*								
(Intercept)	157.54	13.22	11.92	0.00***	189.43	25.18	7.52	0.00***
Female literacy	− 1.21	0.21	− 5.7	0.00***	− 0.70	0.27	− 2.58	0.01*
Female labor force participation rate	− 0.28	0.13	− 2.23	0.03*	− 0.61	0.15	− 3.95	0.00***
Female scheduled caste and tribe	0.33	0.12	2.63	0.01*	0.14	0.10	1.46	0.15
Access to sanitation facilities	0.07	0.12	0.62	0.54	0.12	0.13	0.91	0.36
Access to safe water	0.10	0.13	0.74	0.46	− 0.25	0.20	− 1.25	0.21
Road infrastructure	− 0.40	0.08	− 4.93	0.00***	0.06	− 0.75	0.45	
Access to medical facilities	0.03	0.07	0.46	0.64	− 0.13	0.09	− 1.52	0.13
Urbanization	− 0.10	0.11	− 0.93	0.36	0.14	0.08	1.71	0.09
Access to electricity	− 0.10	0.13	− 0.74	0.46	− 0.43	0.11	− 3.93	0.00***
Adjusted R^2^		0.71			0.62			
*Cluster 5*								
(Intercept)	188.44	36.76	5.13	0.00***	193.82	41.53	4.67	0.00***
Female literacy	− 2.46	0.72	− 3.42	0.00**	− 0.80	0.42	− 1.89	0.07
Female labor force participation rate	0.87	0.6	1.45	0.15	0.81	0.48	1.67	0.10
Female scheduled caste and tribe	− 0.63	0.42	− 1.5	0.14	− 0.62	0.33	− 1.85	0.07
Access to sanitation facilities	− 1.31	0.76	− 1.72	0.09	− 0.46	0.30	− 1.53	0.13
Access to safe water	− 0.84	0.25	− 3.39	0.00**	− 0.48	0.30	− 1.62	0.11
Road infrastructure	0.64	0.48	1.34	0.19	− 0.04	0.09	− 0.50	0.62
Access to medical facilities	− 0.06	0.12	− 0.49	0.62	0.02	0.08	0.30	0.77
Urbanization	0.68	0.52	1.32	0.20	0.24	0.23	1.02	0.31
Access to electricity	1.04	0.38	2.74	0.01**	0.21	0.16	1.28	0.21
Adjusted R^2^		0.54			0.26			
*Cluster 6*								
(Intercept)	132.81	16.97	7.82	0.00***	69.79	23.36	2.99	0.00**
Female literacy	0.24	0.42	0.58	0.57	0.29	0.34	0.85	0.40
Female labor force participation rate	− 0.92	0.35	− 2.62	0.01*	1.02	0.34	3.02	0.00**
Female scheduled caste and tribe	0.28	0.23	1.22	0.23	0.00	0.13	− 0.03	0.98
Access to sanitation facilities	− 1.09	0.50	− 2.19	0.03*	− 0.35	0.17	− 2.02	0.05*
Access to safe water	0.00	0.15	− 0.02	0.99	0.04	0.12	0.32	0.75
Road infrastructure	0.75	0.29	2.56	0.01*	0.10	0.09	1.15	0.26
Access to medical facilities	− 1.03	0.33	− 3.14	0.00**	− 0.29	0.11	− 2.64	0.01*
Urbanization	− 0.30	0.34	− 0.90	0.37	0.32	0.14	2.30	0.03*
Access to electricity	− 0.11	0.39	− 0.28	0.78	− 0.40	0.17	− 2.41	0.02*
Adjusted R^2^		0.45				0.53		

*p < 0.05; **p < 0.01; ***p < 0.001

*Cluster 1* mostly encompasses districts from the southern states of India and districts from Gujarat and Rajasthan in the west. It is characterized by high female literacy rates and the lowest proportion of women in scheduled castes and tribes. It also has the lowest U5MR in both years, with a marginal change over time (78.5 in 1986–1987 and 71.2 in 2006–2007). Yet, the most significant factors (*p* < 0.01) explaining the variation in U5MR in 1991 were female literacy, the share of females in scheduled tribes and castes, and access to paved roads. In 2011, the percentage of females in scheduled castes and tribes and literacy rates were still significantly associated with U5MR. While sanitation was positively related to U5MR in 1991, it was no longer significant in 2011. This could perhaps be linked to the sharp reduction in inequalities in access to sanitation from 1991 to 2011, as shown in the coefficients of variation in “Appendix 2” (CV = 0.82 in 1991 and 0.48 in 2011).

*Cluster 2* mainly represents districts from Uttar Pradesh, Madhya Pradesh, and Chhattisgarh, which have been designated as “High Focus States” by the government of India (along with five other states). This cluster has the highest mortality rates in both the 1991 and 2011 censuses (140.1 in the period 1986–1987 and 94.4 in the period 2006–2007). U5MR has decreased substantially in this cluster. Access to paved roads in 1991 was a highly significant predictor of U5MR in this cluster (*p* < 0.01), while female labor force participation and access to medical facilities were other significant predictors (*p* < 0.05). Female labor force participation in 2011 was still a significant predictor of U5MR in this cluster, along with access to sanitation facilities. The average level of access to sanitation facilities is very low (31%) in this cluster. The insignificant effect of access to roads and medical facilities in 2011 could be related to the significant improvement in the level of these indicators from 1991 to 2011.

*Cluster 3* is also a high-mortality cluster and mainly represents districts from the northeastern states of India. The level of U5MR in this cluster was high in 1991, but progress has been slow. Female literacy was a significant predictor of U5MR in both periods (*p* < 0.001 in 1991 and *p* < 0.05 in 2011). The other significant predictor in 1991 was female labor force participation, while in 2011, it was access to sanitation facilities and the proportion of scheduled castes and tribes that were significant. Despite changes in the mean values of each predictor, the coefficients of variation for each covariate have not changed substantially over the last 20 years—with the exceptions of female literacy and access to sanitation facilities,—thus suggesting that substantial variations persist in this cluster at the level of socioeconomic development.

*Cluster 4* represents the second-lowest U5MR cluster and is comprised of districts mainly from Maharashtra in the west and Himachal Pradesh, Punjab and Haryana in the north. Female agency variables such as female literacy and labor force participation explain most of the variation in U5MR for both of this cluster censuses. The other important predictors of U5MR in 1991 for this cluster were access to paved roads and the proportion of females in scheduled caste and tribes, both of which were replaced by access to electricity in 2011. This is the only cluster where female labor force participation has increased over time. This cluster also contrasts with other clusters in that a large proportion of households have access to electricity.

*Cluster 5* is the second-highest U5MR cluster represented by districts from Bihar and Jharkhand, which are also designated as “High Focus States”. The U5MR variation in 1991 for this cluster is determined mainly by variations in female literacy, access to safe water and access to electricity. However, we found a positive relationship between access to electricity and U5MR. This counter-intuitive result could be related to the fact that this cluster had the smallest percentage of households with access to electricity (For Census 1991—18.3% versus 58.3% in Cluster 4), and there could be additional variables confounding this association. Contrary to the other clusters, we did not find any significant predictor of U5MR in this cluster in 2011, suggesting that there could be important covariates that were left out of our models.

*Cluster 6* represents mainly districts from states in the eastern region of India, such as Jharkhand, Orissa, and West Bengal. The decline in the level of U5MR is substantial, but the coefficient of variation has only marginally decreased ("Appendix 2"). Access to medical facilities along with sanitation and female labor force participation were the common predictors of U5MR in both censuses for this cluster. Female labor force participation was positively associated with U5MR in this region in the second period. The other significant predictors were road infrastructure in 1991 and access to electricity and urbanization in 2011.

## Discussion

Infant and child survival depends on a host of demographic, socioeconomic, and contextual factors, all of which vary locally [32]. Determining the contribution of each of these factors at the smallest geographical level provides useful insights for programs that aim to reduce child mortality. From this study in India, it is evident that each covariate shows specific spatial variations, which cannot be readily summarized in a simple pattern. The spatial heterogeneity in the relationships that U5MR has with socioeconomic, development and environmental health factors (including sign reversals) indicates that applying constant regression coefficients irrespective of geographic location does not adequately describe the relationships between U5MR and the factors that influence it. The maps of *t*-values clearly reveal strong spatial heterogeneity with several clusters of districts where the relationship does not follow the expected direction of the relationship found in the global OLS model. The expected directions of relationships were followed in most districts of India by the proportion of female literates, the share of females belonging to scheduled castes or tribes, and the percentage of villages with access to medical facilities. However, although the negative associations in most districts were consistent with expectations, specifically regarding factors such as the female labor force participation rate and access to safe water, to sanitation facilities, to paved roads and to electricity, few significant pockets were found where the relationship was positive. More research is needed to understand how these unexpected relationships are possible. For example, our indicator of access to “safe” water constitutes access to a hand pump, to a tube well or to tap water, but that includes both treated and untreated tap water. It is possible that tap water in some areas of the country is insufficiently treated and is detrimental to children’s health or that the delivery of tap water is more often interrupted than in households using water wells. A previous study conducted in India found a higher incidence of diarrhea outcomes in children living in households with piped water [33], possibly due to contamination from unclean storage tanks and water distribution networks. Hence, the impact of access to water on child health will also vary with local conditions such as water sources and the reliability of distribution networks.

Health indicators tend to be clustered, and some of this clustering may be linked to shared neighborhood socioeconomic and health characteristics. Cluster analysis helped to categorize such clusters. Six principal clusters were identified with distinct relationships among our main covariates and U5MR based on the local regression coefficients from GWR. These clusters do not follow the regional boundaries suggested by the previous studies. The high mortality states of India that are often typically classified as high focus states [10] were classified in this study into three different clusters based on the relationship of the factors associated with U5MR. The most significant factors associated with U5MR in three high-mortality clusters were female agency variables: female literacy and female labor force participation. Maternal education has been established as the single most important global determinant of differences in child survival [34]. On the other hand, previous study on India [16] argue that female agency variables like female literacy and labor force participation do not play a dominant role in reducing child mortality in the previous years. Our study suggests that the effect of female agency variables is more dominant in areas with high U5MR. Access to safe water, sanitation facilities and electricity in 2011 was the other set of indicators that played a key role in explaining U5MR variations in these three clusters. Previous studies have shown that the basic infrastructure and factors related to facilities are still important in reducing child mortality in Indian states with high mortality [11]. However, this study also highlights that existing socioeconomic or environmental health variables have low explanatory power in high focus states like Bihar and Jharkhand. This suggests that there might be other social, cultural, and economic conditions accounting for the variation in U5MR in Cluster 5.

Clusters 1 and 4 were the two lowest mortality clusters. However, the factors explaining the variations in these two clusters were not the same. While female literacy and labor force participation were significantly associated with U5MR in Cluster 4, the indicators related to facilities were more important in Cluster 1, which is clearly in a more advanced stage of development. A previous study [35] also showed that the states in Cluster 1 have low levels of multidimensional poverty, which covers indicators related to health, knowledge, income, employment, and household environment. An interesting observation for Cluster 1 is that the proportions of females in scheduled tribes or scheduled castes constitute a significant risk factor for U5MR in this cluster, even if it had the lowest proportions of women in these tribes and castes in both census years while simultaneously being the most advanced in terms of economic development. This finding contrasts with an earlier study of the influence of caste on child mortality in India, which concluded that caste differentials in child health were particularly pronounced in the poorer districts of India [36].

Village access to paved roads was one of the prominent determinants of U5MR in both high- and low-mortality clusters in 1991, and access to sanitation facilities in 2011. A decline occurred in the U5MR contribution from access to well-paved roads at the local level, which can be attributed to increasing the accessibility of these facilities at the district level [17].

This study is subject to certain limitations, which point to directions for future work. First, we used indirect methods to generate district-level estimates, although such methods do not allow estimating age-specific mortality rates and thus impede monitoring of neonatal mortality. According to UNICEF, up to 60% of children’s deaths in India were among neonates in 2016 [5]. Generating age-specific mortality rates at the district level requires a more comprehensive approach, one that uses full birth histories collected in large-scale multi-round survey programs such as the District Level Household and Facility Survey and the National Family Health Survey. However, because of sampling and non-sampling errors, the resulting estimates may vary substantially between data sources for a given district; therefore, statistical approaches should be employed to estimate smooth trends in U5MR for all districts and their uncertainty intervals [1]. Second, we constructed our covariates based only on censuses because we needed them to be available for the whole period of observation. There is a need to expand our database by including other covariates, such as those constructed from the various Annual Health Surveys—if any of them are able to cover the current study period. Last, since the 2011 census is the last operation considered here, our results refer to the past and may not reflect recent mortality changes.

Apart from these limitations, this study makes a significant contribution to comprehensively examine spatial pattern of U5MR and its local determinants in India. The spatial methods utilized in this study can potentially contribute to public health research by allowing researchers to better understand the etiology and spatial processes and offer results beyond global models. GWR is identified as one of the geostatistical methods that should be promoted in health studies in light of the locality of health outcomes [37]. Further, this study demonstrates the utility of combining GWR and cluster analysis as a methodological approach to study local level variation in public health indicators. It could be applied in any country using publicly available aggregate level information from census or survey data. There are several other studies [38, 39] in other high mortality countries, which identified the importance of spatial analysis in mortality studies. However, these studies were focused on estimating mortality at the local levels and adjusting global analytical models of mortality for spatial clustering. This study makes a useful contribution in this direction by studying spatial and temporal changes in local level determinants of U5MR in high mortality region and check spatial non-stationarity of relationship between U5MR and its predictors, as spatial non-stationarity can hamper efforts to achieve meaningful interpretation of mortality or public health data. By capturing these spatial and temporal changes in local relationships of public health indicators with its predictors, the authorities can design more effective local strategies. Maps displaying the local GWR coefficients are relatively easy to interpret and could feed into the debates on how to design health interventions at the local level, but also on which specific subpopulations should be targeted.

## Conclusion

The main aims of this study were, first, to examine the effects that a series of socioeconomic, development, and environmental health factors have on U5MR for each district and, second, to study the spatial heterogeneity in each factor’s relationship with U5MR from 1991 to 2011. Considerable geospatial clustering of high mortality districts is observed for both of these census years. After controlling for the effect of other factors in GWR models, we observe coefficients with both positive and negative effects from each factor at the district level. The spatial non-stationary test results also confirm the spatial non-stationarity of these relationships. This justified our use of geographically weighted regression models to study factors associated with U5MR at the district level. Further, this implies local variations in the contribution of each socioeconomic, development and environmental health factor. This, together with the GWR models for different years, implies that they also vary over time. Cluster analysis of GWR coefficients help identify not only the key clusters of districts in India, but also the important predictors associated with each cluster. These clusters do not identically follow the regional boundaries proposed in previous studies. To accelerate U5MR reduction in India, intervention programs should be designed at the local level, and they must consider local-level determinants of U5MR. This would not only accelerate progress towards the SDG target for child mortality in India, but it would also minimize the health and socioeconomic inequalities across the districts in this country.

## Appendix 2

See Table 6.

**Table 6:** Descriptive statistics of independent variables by cluster (including cluster comparison for 2011), 1991 and 2011

	Under five mortality rate	Female literacy	Female labor force participation rate	Female scheduled caste and tribe	Access to sanitation facilities	Access to safe water	Road Infra structure	Access to medical facilities	Urbanization	Access to electricity
*Cluster 1*										
1991										
Mean	78.47	44.48	33.34	21.61	27.15	58.88	60.25	42.83	25.21	53.90
CV	0.37	0.48	0.45	0.62	0.82	0.38	0.44	0.69	0.71	0.39
2011										
Mean	71.19	67.17	30.41	23.31	51.71	79.42	80.53	61.74	33.68	85.13
CV	0.30	0.21	0.38	0.60	0.48	0.27	0.34	0.39	0.59	0.18
*2011**										
Mean	73.22	68.10	30.71	23.65	50.54	78.19	80.08	60.04	34.12	83.87
CV	0.33	0.21	0.36	0.58	0.51	0.29	0.33	0.43	0.64	0.21
*Cluster 2*										
1991										
Mean	140.11	31.24	29.04	31.23	17.09	55.26	29.85	31.52	22.67	42.04
CV	0.24	0.42	0.64	0.40	0.69	0.30	0.44	1.14	0.77	0.48
2011										
Mean	94.37	61.08	25.52	32.24	31.15	83.77	58.92	34.99	26.55	62.34
CV	0.18	0.17	0.47	0.39	0.51	0.13	0.31	0.45	0.59	0.37
2011*										
Mean	93.42	59.40	17.67	24.22	44.05	89.77	61.06	47.29	27.82	56.16
CV	0.22	0.16	0.58	0.50	0.47	0.11	0.42	0.44	0.60	0.47
*Cluster 3*										
1991										
Mean	99.87	42.91	43.88	55.13	39.17	46.82	23.79	21.51	14.90	31.35
CV	0.46	0.31	0.50	0.64	0.52	0.42	0.60	0.83	0.78	0.58
2011										
Mean	86.40	67.59	31.39	55.73	69.19	60.58	30.01	47.28	18.61	54.32
CV	0.27	0.16	0.48	0.64	0.24	0.36	0.73	0.50	0.67	0.42
2011*										
Mean	88.13	65.58	30.28	52.47	61.46	66.73	36.16	42.52	17.97	52.07
CV	0.26	0.17	0.52	0.68	0.41	0.36	0.73	0.59	0.67	0.43
*Cluster 4*										
1991										
Mean	89.73	41.54	23.42	28.53	27.94	72.80	56.39	54.86	25.79	58.31
CV	0.33	0.37	0.93	0.71	0.69	0.26	0.59	0.63	0.77	0.44
2011										
Mean	77.13	67.50	22.15	28.61	62.17	91.26	70.76	50.71	32.10	79.33
CV	0.27	0.15	0.62	0.67	0.28	0.11	0.40	0.44	0.70	0.28
2011*										
Mean	75.44	67.81	27.65	33.65	53.58	86.56	71.12	44.40	30.26	84.45
CV	0.23	0.15	0.50	0.57	0.43	0.13	0.38	0.50	0.66	0.15
*Cluster 5*										
1991										
Mean	118.93	23.10	20.66	27.00	10.09	54.30	30.11	45.19	13.79	18.31
CV	0.28	0.37	0.48	0.55	0.79	0.39	0.32	0.76	0.79	0.84
2011										
Mean	98.38	56.64	15.33	27.47	23.67	88.07	66.37	40.09	13.57	32.06
CV	0.14	0.12	0.41	0.51	0.51	0.16	0.32	0.57	0.78	0.62
2011*										
Mean	89.38	55.39	13.94	25.69	23.85	84.19	72.01	46.69	14.14	25.85
CV	0.11	0.10	0.39	0.57	0.35	0.23	0.31	0.43	0.77	0.66
*Cluster 6*										
1991										
Mean	101.40	31.96	20.44	30.42	17.24	58.96	28.82	22.69	18.57	23.56
CV	0.30	0.45	0.59	0.50	0.96	0.43	0.48	0.62	0.95	0.76
2011										
Mean	78.91	60.18	16.05	31.53	35.28	81.39	64.09	40.44	20.07	41.77
CV	0.21	0.20	0.43	0.49	0.70	0.21	0.33	0.45	0.89	0.61
2011*										
Mean	82.52	61.39	17.62	34.16	39.20	79.76	55.86	42.75	19.05	45.41
CV	0.25	0.19	0.48	0.55	0.67	0.23	0.39	0.48	0.91	0.57

*These values are based on clusters formed from *t*-values of GWR in the 2011 census

## References

[1] Alkema L, New JR (2014). Global estimation of child mortality using a bayesian b-spline bias-reduction. Ann Appl Stat.

[2] Hill K, You D, Inoue M, Oestergaard M, Technical Advisory Group Members (2012). Child mortality estimation: accelerated progress in reducing global child mortality, 1990–2010. PLoS Med.

[3] Rajaratnam J, Linda T, Lopez A, Murray C, Rajaratnam J, Linda T, Lopez A, Murray C (2010). Measuring under-five mortality: validation of new low-cost methods. PLoS Med.

[4] United Nations General Assembly. Transforming our world: the 2030 Agenda for sustainable development, Annex to A/69/L.85. http://www.un.org/ga/search/view_doc.asp?symbol=A/69/L.85&Lang=E. Accessed 22 Apr 2018.

[5] UN IGME (United Nations Inter-Agency Group for Child Mortality Estimation). Levels and trends in child mortality: 2017 Report, UNICEF-WHO-WB-UNPD. http://www.childmortality.org. Accessed 22 Apr 2018.

[6] Tabutin D, Masquelier B (2017). Mortality inequalities and trends in low- and middle-income countries. Population.

[7] Registrar General of India. SRS statistical report 2015. http://www.censusindia.gov.in/vital_statistics/SRS_Reports_2015.html. Accessed 15 May 2018.

[8] Singh A, Pathak PK, Chauhan RK, Pan W (2011). Infant and child mortality in India in the last two decades: a geospatial analysis. PLoS ONE.

[9] Ram U, Jha P, Gerland P, Hum RJ, Rodriguez P, Suraweera W (2015). Age-specific and sex-specific adult mortality risk in India in 2014: analysis of 0.27 million nationally surveyed deaths and demographic estimates from 597 districts. Lancet Glob Health.

[10] Kumar C, Singh PK, Rai RK (2012). Under-five mortality in high focus states in India: a district level geospatial analysis. PLoS ONE.

[11] Gupta AK, Ladusingh L, Borkotoky K (2016). Spatial clustering and risk factors of infant mortality: district-level assessment of high focus states in India. Genus.

[12] Dandona L (2017). Nations within a nation: variations in epidemiological transition across the states of India, 1990–2016 in the global burden of disease study. The Lancet.

[13] Raj A, Saggurti N, Winter M, Labonte A, Decker MR, Balaiah D (2010). The effect of maternal child marriage on morbidity and mortality of children under 5 in India: cross sectional study of a nationally representative sample. BMJ.

[14] Ram U, Jha P, Ram F, Kumar K, Awasthi S, Shet A (2013). Neonatal, 1–59 month, and under-5 mortality in 597 Indian districts, 2001 to 2012: estimates from national demographic and mortality surveys. Lancet Glob Health.

[15] Guilmoto CZ, Saikia N, Tamrakar V, Bora JK (2018). Excess under-5 female mortality across India: a spatial analysis using 2011 census data. Lancet Glob Health.

[16] Bhattacharya PC (2006). Economic development, gender inequality, and demographic outcomes: evidence from India. Popul Dev Rev.

[17] Bhattacharya PC, Chikwama C (2011). Inequalities in child mortality in India: a district-level analysis. Asian Popul Stud.

[18] Griffiths P, Madise N, Whitworth A, Matthews Z (2004). A tale of two continents: a multilevel comparison of the determinants of child nutritional status from selected African and Indian regions. Health Place.

[19] Choudhary PK (2015). Explaining the role of parental education in the regional variations in infant mortality in India. Asia Pac Policy Stud.

[20] Arokiasamy P (2004). Regional patterns of sex bias and excess female child mortality in India. Population.

[21] Brass W, Coale AJ, Brass W, Coale AJ, Demeny P, Heisel DF (1968). Methods of analysis and estimation. The demography of tropical Africa.

[22] Hill K, Moultrie T, Dorrington R, Hill A, Hill K, Tim I, Zaba B (2013). Indirect estimation of child mortality. Tools for demographic estimation.

[23] Anselin L, Bera AK, Ullah A, Giles DEA (1998). Spatial dependence in linear regression models with an introduction to spatial econometrics. Handbook of applied economic statistics.

[24] Kutner M, Nachtsheim C, Neter J (2004). Applied linear statistical models.

[25] Wang D, Chi G (2017). Different places, different stories: a study of the spatial heterogeneity of county level fertility in China. DemRes.

[26] Fotheringham AS, Brunsdon C, Charlton ME (2003). Geographically weighted regression: the analysis of spatially varying relationships.

[27] Wheeler D, Tiefelsdorf M (2005). Multicollinearity and correlation among local regression coefficients in geographically weighted regression. J Geogr Syst.

[28] Leung Y, Mei CL, Zhang WX (2002). Statistical tests for spatial nonstationarity based on the geographically weighted regression model. Environ Plan A.

[29] Wimberly MC, Yabsley MJ, Baer AD, Dugan VG, Davidson WR (2008). Spatial heterogeneity of climate and land-cover constraints on distributions of tick-borne pathogens. Glob Ecol Biogeogr.

[30] Windle MJS, Rose GA, Devillers R, Fortin MJ (2010). Exploring spatial non-stationarity of fisheries survey data using geographically weighted regression (GWR): an example from the Northwest Atlantic. ICES J Mar Sci.

[31] Kassambara A. Practical guide to cluster analysis in R. STHDA; 2017.

[32] Chin B, Montana L, Basagan X (2011). Spatial modeling of geographic inequalities in infant and child mortality across Nepal. Health Place.

[33] Khanna G. The impact of child health from access to water and sanitation and other socioeconomic factors. HEI Working Paper No: 02/2008 Graduate Institute of International studies, Geneva. 2008. Error! Hyperlink reference not valid.https://ideas.repec.org/p/gii/giihei/heidwp02-2008.html. Accessed 20 June 2018.

[34] Fuchs R, Pamuk E, Lutz W (2010). Education or wealth: Which matters more for reducing child mortality in developing countries?. Vienna Yearb Popul Res.

[35] Dehury B, Mohanty SK. Regional estimates of multidimensional poverty in India. Economics Discussion Papers, No 2015-34, Kiel Institute for the World Economy. 2015. http://www.economics-ejournal.org/economics/discussionpapers/2015-34. Accessed 15 May 2018.

[36] Dommaraju P, Agadjanian V, Yabiku S (2008). The pervasive and persistent influence of caste on child mortality in India. Popul Res Policy Rev.

[37] Young LJ, Gotway CA, Atkinson PM, Lloyd CD (2010). Using geostatistical methods in the analysis of public health data: the final frontier?. geoENV VII—geostatistics for environmental applications.

[38] Burke M, Heft-Neal S, Bendavid E (2016). Sources of variation in under-5 mortality across sub-Saharan Africa: a spatial analysis. Lancet Glob Health.

[39] Pezzulo C, Bird T, Utazi EC, Sorichetta A, Tatem AJ, Yourkavitch J, Burgert-Brucker CR. Geospatial modeling of child mortality across 27 countries in sub-Saharan Africa. DHS Spatial Analysis Reports No. 13. Rockville, Maryland, USA: ICF International; 2016.

